# Relationship between Daily and In-laboratory Gait Speed among Healthy Community-dwelling Older Adults

**DOI:** 10.1038/s41598-019-39695-0

**Published:** 2019-03-05

**Authors:** Naoto Takayanagi, Motoki Sudo, Yukari Yamashiro, Sangyoon Lee, Yoshiyuki Kobayashi, Yoshifumi Niki, Hiroyuki Shimada

**Affiliations:** 1 Tokyo Research Laboratories, Kao Corporation, 2-1-3 Bunka, Sumida-ku, Tokyo, 131-8501 Japan; 2 Department of Preventive Gerontology, Center for Gerontology and Social Science, National Center for Geriatrics and Gerontology, 7-430 Morioka, Obu, Aichi 474-8511 Japan; 3 Exercise motivation and Physical function Augmentation Research Team, Human Augmentation Research Center, National Institute of Advanced Industrial Science and Technology, Waterfront 3F, 2-3-26, Aomi, Koto-ku, Tokyo, 135-0064 Japan

## Abstract

Gait speed in laboratory settings (in-laboratory gait speed) is one of the important indicators associated with the decline in functional abilities in older adulthood. Recently, it has become possible to measure gait speed during daily living (daily gait speed) using accelerometers. However, the relationship between these two gait speed parameters is unclear. This study aimed to compare in-laboratory gait speed, measured by a sheet-type pressure sensor, and daily gait speed, measured by an accelerometer, in healthy community-dwelling older adults. Participants were aged ≥60 years, residing in Takahama city, Aichi, Japan. To calculate daily gait speed, participants were instructed to wear a tri-axial accelerometer on their waist. A total of 1965 participants were included in the final analysis. The results showed a weak association (*r* = 0.333, *p* < 0.001) between the two gait speed parameters. Furthermore, average daily gait speed was significantly lower than average in-laboratory gait speed. However, both gait speed parameters declined significantly with age. These results suggest that, in addition to in-laboratory gait speed, daily gait speed may be a helpful parameter for predicting decline in functional abilities.

## Introduction

Gait speed is one of the important indicators of the decline in an individual’s functional abilities, especially in older adults^1–3^. Previous studies reported that slow gait speed is associated with a loss of maintenance of instrumental activities of daily living (IADL)^2^, mild cognitive impairment (MCI)^4^, and the risk of cardiovascular death^5^. Therefore, gait speed assessment in older adults can help predict the decline in their functional abilities.

Traditionally, gait speed has been measured and assessed mainly in laboratory settings by using a stopwatch, a tape measure, and a sheet-type pressure sensor^6,7^. Recently, it has become possible to assess gait speed in daily living (in free-living conditions) by using wearable sensors. Multiple researchers have assessed daily gait speed using a tri-axial accelerometer^8–11^.

Although various studies have reported the methodology for and accuracy of gait speed measurements using wearable sensors, the relationship between daily gait speed and that in laboratory settings (in-laboratory gait speed) remains unclear. Previous studies reported that both gait speed parameters declined with age^12,13^. However, these two gait speed parameters were measured in different participants. Therefore, to clarify the relationship between the two gait speed parameters, it is essential to measure these within the same participants.

Accordingly, the present study aimed to measure daily gait speed using a tri-axial accelerometer, and in-laboratory gait speed using a sheet-type pressure sensor, among healthy community-dwelling older adults. This was done to confirm if these parameters decline with age. Another purpose was to compare between these parameters within the same participants. Daily gait speed has been measured continuously during gait in free-living conditions. However, because in-laboratory gait speed has only been measured several times in one measurement day, some participants may walk faster or more carefully during such assessments than in free-living conditions. Therefore, we hypothesized that these two gait speed parameters would be inconsistent, because although participants may not be able to change their daily gait speed, they may be able to change their in-laboratory gait speed intentionally.

## Results

Table 1 shows the demographic results and average step counts of participants in the different age groups. Significant differences between the age groups were found for age (*p* < 0.001, *η*^2^ = 0.826), height (*p* < 0.001, *η*^2^ = 0.055), weight (*p* < 0.001, *η*^2^ = 0.035), and average steps per day (*p* < 0.001, *η*^2^ = 0.064).

**Table 1:** Demographics of participants and average steps for each age group.

	All (n = 1965)	60–69 years (n = 1013)	70–79 years (n = 775)	≥ 80 years (n = 177)	Significant difference
Age (years)	70.3 ± 6.3	65.2 ± 2.6	74.1 ± 2.7	82.6 ± 2.6	*p < 0.001 *η*^2^ = 0.826
Sex (males: females)	766: 1199 [39%: 61%]	396: 617 [39%: 61%]	304: 471 [39%: 61%]	66: 111 [37%: 63%]	*χ*^2^ = 0.238 *p* = 0.888 *V* = 0.011
Height (cm)	156.3 ± 8.5	158.0 ± 8.2	155.1 ± 8.4	151.5 ± 8.5	*p < 0.001 *η*^2^ = 0.055
Weight (cm)	57.4 ± 10.2	58.9 ± 10.7	56.5 ± 9.4	52.4 ± 8.9	*p < 0.001 *η*^2^ = 0.035
BMI (kg/m^2^)	23.4 ± 3.2	23.5 ± 3.3	23.4 ± 3.0	22.8 ± 3.1	*p* = 0.021 *η*^2^ = 0.004
Average steps (steps/day)	6478.7 ± 3021.3	7062.5 ± 2987.3	6183.6 ± 2947.9	4429.8 ± 2402.1	*p < 0.001 *η*^2^ = 0.064

Data are shown as the mean ± SD. One-way ANOVAs or *χ*^2^ tests were conducted to compare age groups. *p < 0.05, *η*^2^ > 0.01.

Table 2 compares the two gait speed parameters between age groups and measurement methods. Significant main group effects were found for age group (*p* < 0.001, *η*^2^ = 0.135) and measurement method (*p* < 0.001, *η*^2^ = 0.042). However, no interaction effect was found (*p* = 0.260, *η*^2^ < 0.001).

**Table 2:** Comparison of 6.4-m gait speed and daily gait speed between age groups.

	All (n = 1965)	60–69 years (n = 1013)	70–79 years (n = 775)	≥ 80 years (n = 177)	Main effects		Interaction effect
					Age	Measurement	Age × Measurement
6.4-m gait speed (cm/s)	117.0 ± 19.9	122.3 ± 18.3	114.0 ± 19.2	100.6 ± 20.9	*p < 0.001 *η*^2^ = 0.135	*p < 0.001 *η*^2^ = 0.042	*p* = 0.260 *η*^2^ < 0.001
Daily gait speed (cm/s)	109.7 ± 22.6	115.2 ± 23.0	105.8 ± 21.2	95.6 ± 14.9			

Data are shown as the mean ± SD. Two-way repeated-measures ANOVAs were conducted. *p < 0.05, *η*^2^ > 0.01.

**Figure 1:**
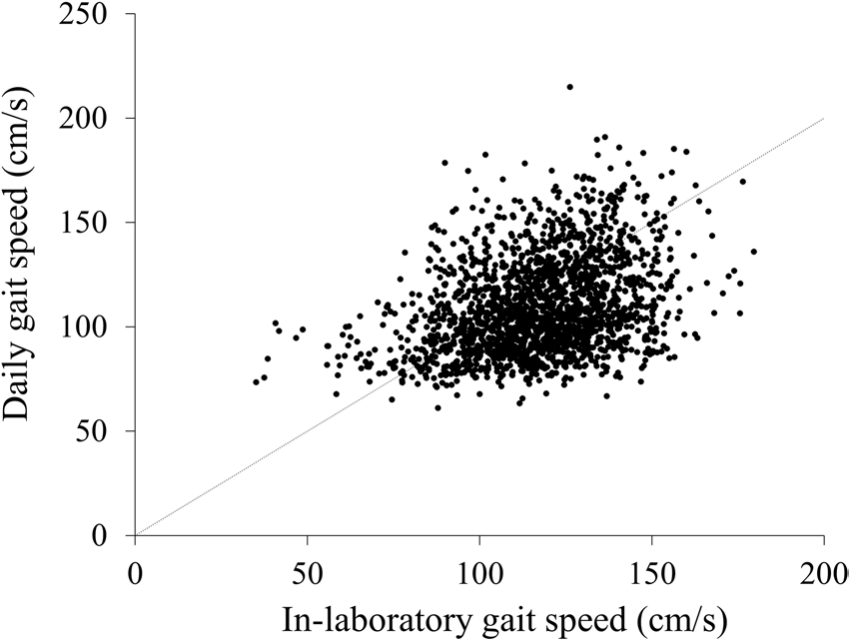
Relationship between daily and in-laboratory gait speed. The Pearson’s correlation analysis was used to determine the relationship between the two gait speed parameters. *r* = 0.333, *p* < 0.001; N = 1965.

Figure 1 shows the relationship between daily and in-laboratory gait speed. These two gait speed parameters were positively correlated (*r* = 0.333, *p* < 0.001).

## Discussion

This study aimed to compare daily gait speed, measured by tri-axial accelerometers, and in-laboratory gait speed among healthy community-dwelling older adults. We hypothesized that these two gait speed parameters would be inconsistent, because although participants may not be able to change their daily gait speed, they may be able to change their in-laboratory gait speed intentionally. Findings revealed only a weak association between daily and in-laboratory gait speed (*r* = 0.333, *p* < 0.001). Additionally, the average daily gait speed was significantly lower than the average in-laboratory gait speed. These results support our hypothesis that the two gait speed parameters are inconsistent.

In the present study, we measured daily gait speed across 14 days, and the criteria for inclusion in the analysis were wearing the accelerometer for a total duration of ≥7 days, for ≥10 hours/day. This standard was used in previous studies^14,15^ to measure the total amount of activity in a day. This enabled us to obtain a daily gait speed measure which is not affected by the time and the day. On the other hand, in-laboratory gait speed was measured on one measurement day, where it may be easier for subjects to change their gait speed intentionally. This may explain the lack of a strong association between these parameters in the present study.

The present findings also showed that the average daily gait speed was significantly lower than that in the laboratory. Multi-tasking during walking in daily living conditions could explain this result. A previous study on older adults reported that the average gait speed in laboratory settings with dual tasks (a cognitive task) was significantly slower than that with no task^16^. In daily living, people sometimes walk while performing other tasks. This may be one of the reasons why daily gait speed was significantly lower than in-laboratory gait speed.

Another previous study reported that the usual gait speed is affected by psychological factors^17^. This study showed that the ‘body pain’ and ‘vitality’ scores in the 12-item Short-Form Health Survey (SF-12; Health Related Quality of Life scale) were independent predictors of six-metre gait speed (i.e. in-laboratory gait speed) in older adults^17^. In particular, the body pain score changes depending on the individual’s condition on that day. Some participants may walk faster than usual while enduring the pain in laboratory settings. On the other hand, it may be difficult for participants with body pain to walk faster than usual at all times in daily living. Future studies need to clarify the association between daily gait speed and these factors.

Although a significant difference was observed between the two gait speed parameters, the present study showed that both these parameters declined with age. Multiple previous studies have reported a significant decline in in-laboratory gait speed with age^12,18,19^. However, for daily gait speed, Schimpl *et al*.^13^ only reported the measurement of daily gait speed in healthy participants aged 17–65 years, finding a slight decline of this parameter with increasing age. In the present study, the daily gait speed of 1965 healthy community-dwelling older adults aged over 60 years was measured and a decline with age, similar to that found for in-laboratory gait speed, was observed. These results suggest that, as for in-laboratory gait speed, daily gait speed may be a parameter associated with the decline in functional abilities in older adults. This finding may provide a good basis for the assessment of functional decline in free-living conditions.

Many previous studies reported that in-laboratory gait speed is associated with the decline in an individual’s functional abilities^1–4^. Cesari *et al*. reported that a usual gait speed in laboratory settings of less than 100 cm/s indicates a high risk of negative health-related outcomes in well-functioning older adults^20^. However, while the number of participants whose in-laboratory gait speed was less than 100 cm/s was 381 (19.4%), that of participants with a daily gait speed of less than 100 cm/s was 779 (39.6%). These points suggest that standard expected values or values indicating high-risk individuals need to be determined separately for daily and in-laboratory gait speed, to predict decline in functional abilities more accurately. Future studies on the relationships between daily gait speed and individuals’ functional decline are needed.

Because daily gait speed has been measured by the accelerometer in daily living, this gait parameter can be assessed frequently. In comparison, it is difficult to measure in-laboratory gait speed continuously. High frequency measurements enable the detection of changes in parameters at earlier stages. Therefore, the continuous measurement of daily gait speed may be useful to detect the decline in the functional abilities of older adults.

The present study focused on daily gait speed. Recently, some other gait parameters derived from walking acceleration signal have also been assessed. For instance, Ho *et al*. proposed a method for step length estimation at various gait speeds by using a smartphone^21^. Additionally, Manor *et al*. suggested a smartphone-based assessment of stride time during gait in non-laboratory settings^22^. These gait parameters have been associated with aging^23,24^. Therefore, it may be possible to detect the decline in functional abilities more accurately by using daily gait speed and these parameters together.

There are several limitations in this study. First, the type of shoes worn during gait measurements were not considered. Second, the accelerometer has an LCD screen and participants could see their step counts. Therefore, the amount of activity during the assessment period might have been higher relative to that on ordinary free-living days^25^.

In the present study, we compared daily gait speed, measured by tri-axial accelerometers, and in-laboratory gait speed, measured by a sheet-type pressure sensor, in healthy community-dwelling older adults. The results showed only a weak association between daily and in-laboratory gait speed. Additionally, daily gait speed was significantly lower than that observed in the laboratory. However, both these parameters declined with age. These results suggest that daily gait speed may be a useful parameter for the prediction of functional decline among healthy community-dwelling older adults in free-living conditions.

## Methods

### Participants

The present study was based on data from the Takahama Study of Health Promotion for Older Adults conducted from September 2015 to June 2016. This study is a part of the National Center for Geriatrics and Gerontology Study of Geriatric Syndromes (NCGG-SGS), which is a cohort study that aimed to establish a screening system for geriatric syndromes^26^. The inclusion criteria for this study were age ≥60 years and residence in Takahama city, Aichi, Japan. A total of 4072 community-dwelling older adults participated and agreed to wear the accelerometer during this study. All participants provided written informed consent by reading and signing a consent form that was approved by the institutional review board. This study was carried out in accordance with the guidelines proposed in the Declaration of Helsinki, and the study protocol was approved by the research ethics committee of the National Center for Geriatrics and Gerontology (Approval Number 861).

### Daily data collection and analysis

The participants were instructed to wear the tri-axial accelerometer (HW-100, Kao Corporation, Tokyo, Japan) on their waist at all times while awake, except during swimming or bathing, and to maintain their usual activities. Furthermore, they were instructed to visit one of 75 designated places named as KENKOJISEICHI^14^ in Takahama city, once in 30–40 days, where the data measured by the accelerometer were downloaded onto a tablet computer using a near field communication (NFC) system (RC-380, Sony Corporation, Tokyo, Japan). KENKOJISEICHI included public facilities, gyms, drug stores, cafeterias, and beauty salons, in order to make it as easy as possible for the participants to visit a location and have their data downloaded.

Individuals were excluded if: (i) they did not visit KENKOJISEICHI within 60 days of the date of instruction to begin wearing the accelerometer (n = 1148); (ii) in-laboratory gait speed measurement was not conducted within the Takahama Study of Health Promotion for Older Adults (n = 1); and (iii) participants were unable to meet the criteria for analysis of accelerometer data (n = 958). The criteria for analysis were wearing the accelerometer on their waist for a total duration of ≥7 days, for ≥10 hours/day, during the first 14 days after the day they began wearing the accelerometer. The remaining 1965 participants (48.3%) were included in the final analysis.

### Daily gait speed measurement

Daily gait speed was measured using an accelerometer (HW-100) for continuous monitoring during daily living. HW-100 is a tri-axial accelerometer providing 40 days of continuous recording at a sampling frequency of 64 Hz^14^. This device detects step cycle during gait ranging from 70 to 160 steps/min by medio-lateral and vertical acceleration. It commences recording of tri-axial acceleration during the gait cycle if the measured acceleration of the current cycle and the preceding two cycles are within 10% of one another. Therefore, the accelerometer records tri-axial acceleration during steady-state periods of gait. The generic structure of a gait speed estimation method is as follows: (1) the accelerometer is attached to the body and measures the acceleration signals; (2) with these measurements, mathematical models can be used to estimate gait speed^27^. In the case of the HW-100, daily gait speed was calculated with a model that used composite acceleration during one gait cycle from the tri-axial acceleration measurements. The estimation accuracy of gait speed for this device is shown in Supplementary Fig. S1. Based on the accuracy evaluation conducted in the present study, as evident from Supplementary Fig. S1, the systematic error for this device was 14.3 cm/s, which suggests that this device may have underestimated daily gait speed as compared with the gold standard. Therefore, in the present study daily gait speed was adjusted by adding 14.3 cm/s to the observed value. An average daily gait speed for valid days during a 14-day period was obtained for each participant.

**Figure 2:**
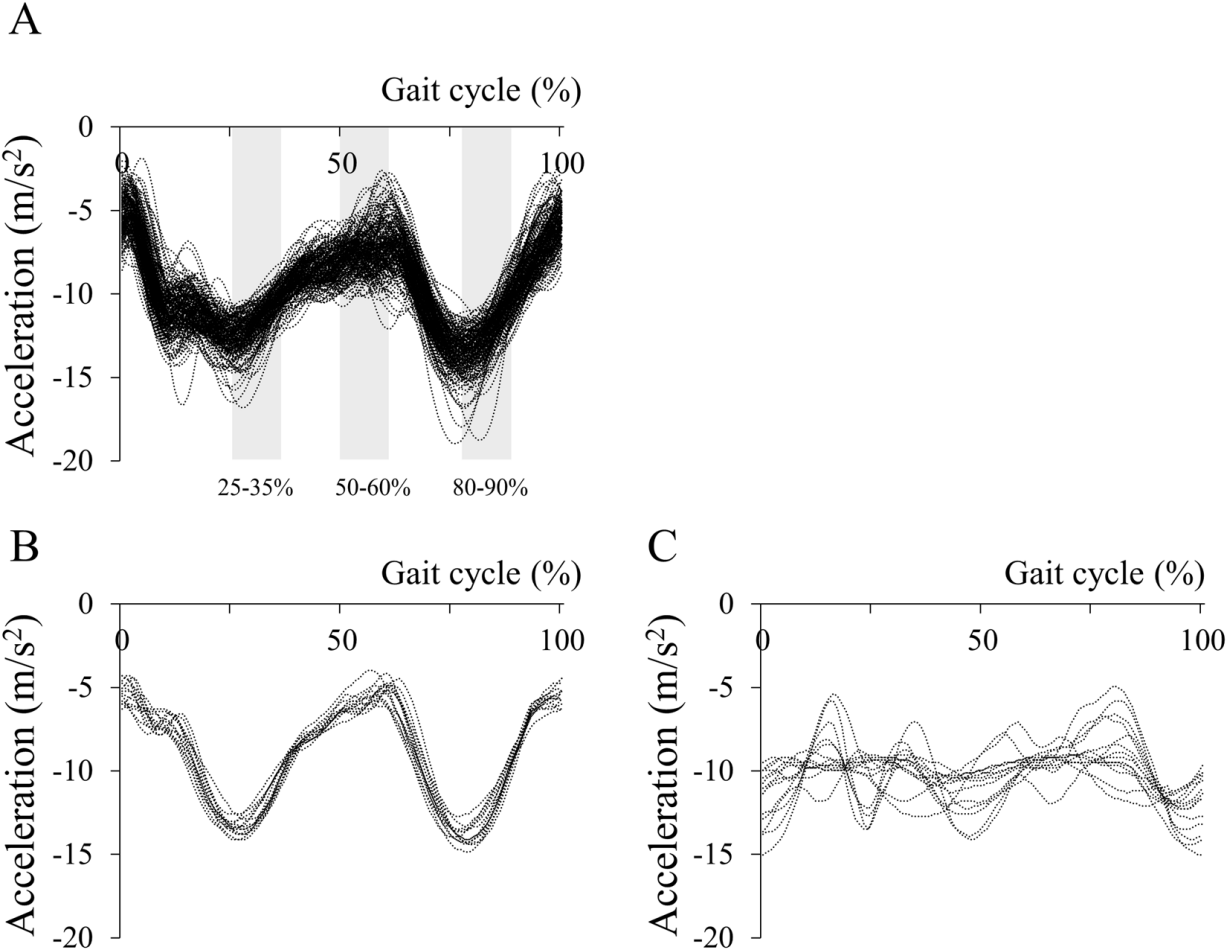
Accelerometer data from the Advanced Industrial Science and Technology (AIST) gait database and from when the device was correctly or incorrectly attached at the waist of the participants. (**A**) Vertical acceleration data for the right waist (anterior superior iliac spine) of 204 participants aged 20 to 77 from the AIST gait database^28^. (**B**) Example data for one participant with the accelerometer attached at the waist for all 14 days. (**C**) Data from another participant with no valid measurement days during the 14-day period. Based on the AIST gait database, if the average acceleration during 25–35% and 80–90% of the gait cycle is greater than that during 50–60% of the gait cycle, the acceleration data are excluded from analysis.

The HW-100 accelerometer also measured step counts and wearing time^14^. For inclusion in the present study, a valid day was defined as a day on which the accelerometer was worn for ≥10 hours^14,15^. Data from the Advanced Industrial Science and Technology (AIST) gait database^28^ were used to define days on which the accelerometer was not correctly worn on the waist, to enable exclusion from analysis. This procedure is illustrated in Fig. 2, which shows the vertical acceleration data from 204 individuals in the AIST gait database and examples of data from present participants who wore the accelerometer correctly and incorrectly.

### In-laboratory gait speed measurement

In the present study, 6.4-m gait speed was defined as in-laboratory gait speed based on a previous study^29^. This parameter was measured using a sheet-type pressure sensor (2.4-m long; Walk Way, Anima Corporation, Tokyo, Japan) placed in the middle of a 6.4-m walkway, operating at a sampling frequency of 100 Hz^7^. The participants were instructed to walk along the walkway at a comfortable pace. They repeated the 6.4-m walk five times and the average in-laboratory gait speed was calculated.

### Statistics

One-way measures analysis of variance (ANOVA) and the *χ*^2^ test were conducted to analyse physical characteristics and average steps per day between different age groups (60–69 years, 70–79 years, 80 years and over). A two-way repeated measures ANOVA was used to assess the main and interaction effects between age groups (60–69 years, 70–79 years, 80 years and over), and gait speed parameters (daily and in-laboratory gait speed). Furthermore, the relationship between the two gait speed parameters was examined by calculating Pearson’s correlation coefficients (*r*). The differences in the means were considered statistically significant if the *p* values were less than 0.05, the partial *η*^2^ values were greater than 0.01, and the *V* values were greater than 0.10^30^. All statistical analyses were performed using the SPSS statistical software package (IBM SPSS Statistics Version 23, SPSS Inc., Chicago, IL, USA).

## Supplementary information

Supplementary Material

## Data Availability

The datasets generated and/or analysed during the current study are not publicly available due to intellectual property reasons, but are available on reasonable request.
