# Supplementary material for: Relationship between Daily and In-laboratory Gait Speed among Healthy Community-dwelling Older Adults
Source: Sci Rep. 2019 Mar 5;9:3496. doi: 10.1038/s41598-019-39695-0 (PMC6401058; doi:10.1038/s41598-019-39695-0)
Supplement: Supplementary file 1 — Supplementary Material [file 41598_2019_39695_MOESM1_ESM.docx]

**Supplementary materials**

**Relationship between Daily and In-laboratory Gait Speed among Healthy Community-dwelling Older Adults**

**Naoto Takayanagi**^1*^**, Motoki Sudo**^1^**, Yukari Yamashiro**^1^**, Sangyoon Lee**^2^**, Yoshiyuki Kobayashi**^3^**, Yoshifumi Niki**^1^**, Hiroyuki Shimada**^2^

^1^Tokyo Research Laboratories, Kao Corporation, 2-1-3 Bunka, Sumida-ku, Tokyo 131-8501, Japan

^2^Department of Preventive Gerontology, Center for Gerontology and Social Science, National Center for Geriatrics and Gerontology, 7-430 Morioka, Obu, Aichi 474-8511, Japan

^3^Digital Human Research Group, Human Informatics Research Institute, National Institute of Advanced Industrial Science and Technology, Waterfront 3F, 2-3-26, Aomi, Koto-ku, Tokyo 135-0064, Japan

^*^takayanagi.naoto@kao.com

**Accuracy evaluation for gait speed measured by the accelerometer**

Walking data were obtained from 22 men and 24 women aged 25 to 59 years. The mean age (± SD) was 40.8 ± 10.9 years; mean height was 165.5 ± 8.7 cm; mean body weight was 59.2 ± 10.3 kg; and mean body mass index (BMI) was 21.5 ± 2.8 kg/m^2^. All the participants were able to walk independently without assistive devices (e.g., canes, crutches, or orthotic devices). The methods and procedures of this study were examined and approved by the Human Research Ethics Committee of the Kao Corporation (No. S143-180129) and adhered to the guidelines of the International Declaration of Helsinki. Written, informed consent was obtained from each participant after they were provided with detailed information about the study.

Gait speed measurement was conducted on a 26-m walkway including 3-m for acceleration and another 3-m for deceleration. Thus, gait speed was measured at the 20-m position in the middle of walkway. Actual gait speed was measured using a stopwatch as a gold standard. Estimated gait speed was measured using the tri-axial accelerometer (HW-100, Kao Corporation, Tokyo, Japan). The participants were instructed to wear this accelerometer on the right side of their waist. They were instructed to walk along the walkway at three kinds of pace; 1) usual pace, 2) faster than usual pace, 3) slower than usual pace. Gait speed at each pace was measured twice. Thus, data from six trials were obtained for each participant.

The relationship between actual and estimated gait speed was examined by calculating Pearson’s correlation coefficients (*r*). The differences in the means were considered statistically significant if the *p* values were less than 0.05. Furthermore, the systematic error (SE) measured in the two different conditions was calculated as an index to evaluate the performance of this accelerometer, as shown in Supplementary Equation 1, below.

*SE* = $\frac{1}{N} \sum_{i=1}^{N} (Actual gait speed-Estimated gait speed)$ 1

The statistical analyses were performed using the SPSS statistical software package (IBM SPSS Statistics Version 23, SPSS Inc., Chicago, IL, USA).

Supplementary Figure S1 shows the relationships between actual and estimated gait speed. There was a strong positive correlation between these parameters (*r* = 0.848, *p* < 0.001). SE between actual and estimated gait speed was 14.3 cm/s (8.9%).


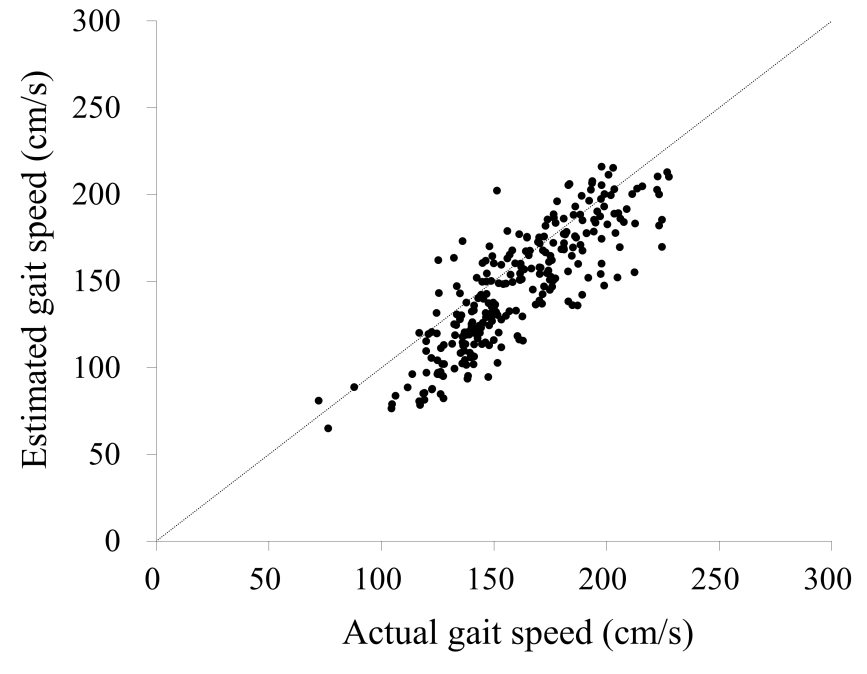


**Figure S1.** Accuracy evaluation for gait speed measured by the tri-axial accelerometer. Pearson’s correlation analysis was used to determine the relationship between the actual gait speed measured using a stopwatch, and estimated gait speed measured using the tri-axial accelerometer (HW-100). Participants were instructed to walk along the walkway at three kinds of pace; 1) usual pace, 2) faster than usual pace, 3) slower than usual pace. Gait speed measurements at each pace were conducted twice. *r* = 0.848, *p* < 0.001; 46 participants × 6 trials.
